# Supplementary material for: Complex‐centric proteome profiling by SEC‐SWATH‐MS
Source: Mol Syst Biol. 2019 Jan 14;15(1):e8438. doi: 10.15252/msb.20188438 (PMC6346213; doi:10.15252/msb.20188438)
Supplement: Supplementary file 8 — Dataset EV7 [file MSB-15-e8438-s008.zip › feature_plots_string/Q86Y33.pdf]

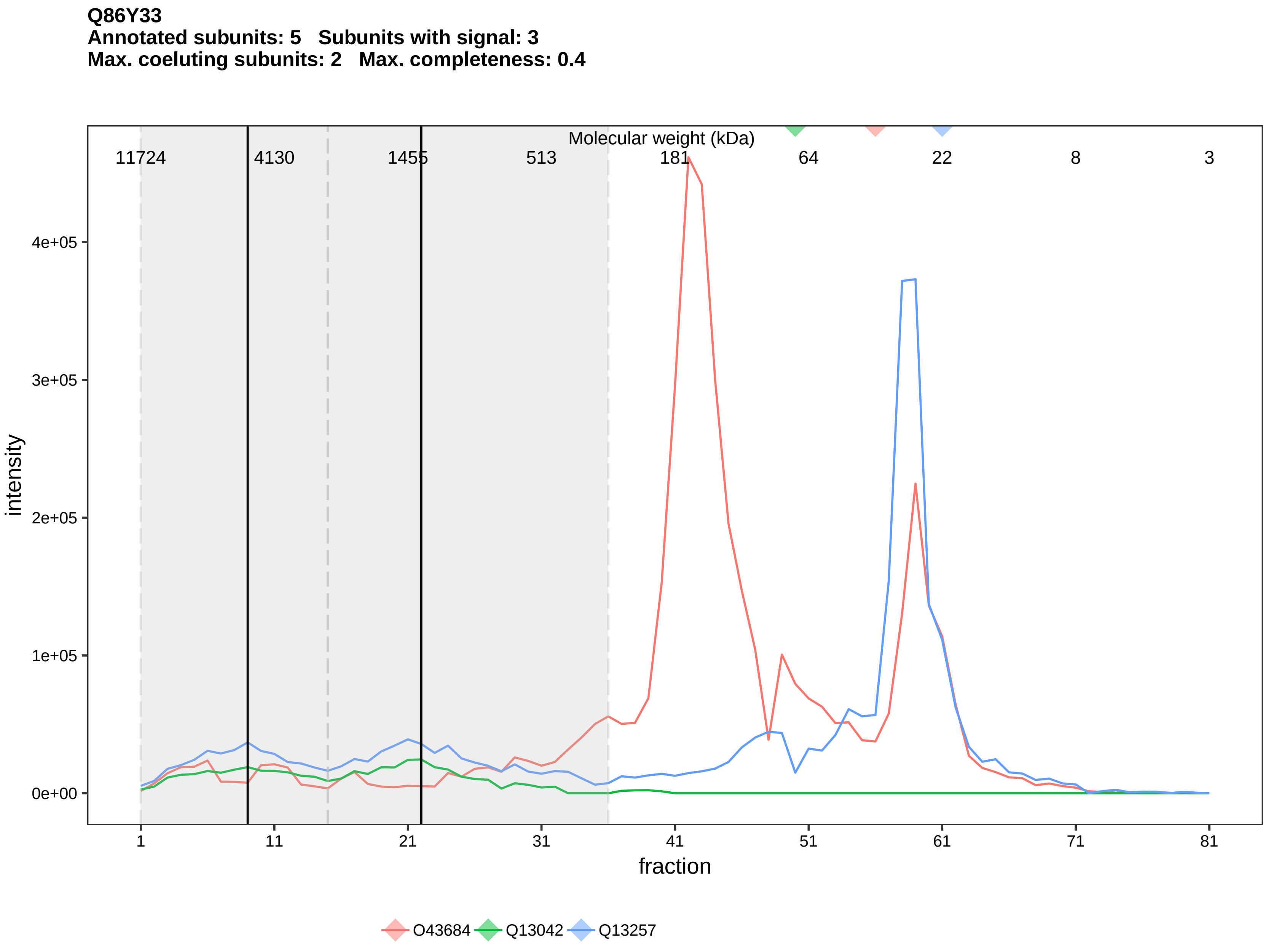
Q86Y33
Annotated subunits: 5 Subunits with signal: 3
Max. coeluting subunits: 2 Max. completeness: 0.4
Molecular weight (kDa)
11724
4130
1455
513
181
64
22
8
3
intensity
4e+05
3e+05
2e+05
1e+05
0e+00
1
11
21
31
41
51
61
71
81
fraction
O43684
Q13042
Q13257
